# Evaluation of Carbohydrates in Natural and Cultured *Cordyceps* by Pressurized Liquid Extraction and Gas Chromatography Coupled with Mass Spectrometry

**DOI:** 10.3390/molecules15064227

**Published:** 2010-06-11

**Authors:** Jia Guan, Feng-Qing Yang, Shao-Ping Li

**Affiliations:** 1 Institute of Chinese Medical Sciences, University of Macau, Macao, China; 2 Department of Pharmaceutics, College of Chemistry and Chemical Engineering, Chongqing University, Chongqing, China

**Keywords:** *Cordyceps*, carbohydrates, polysaccharides, pressurized liquid extraction (PLE), GC-MS

## Abstract

Free and polymeric carbohydrates in *Cordyceps*, a valued edible mushroom and well-known traditional Chinese medicine, were determined using stepwise pressurized liquid extraction (PLE) extraction and GC-MS. Based on the optimized PLE conditions, acid hydrolysis and derivatization, ten monosaccharides, namely rhamnose, ribose, arabinose, xylose, mannose, glucose, galactose, mannitol, fructose and sorbose in 13 samples of natural and cultured *Cordyceps* were qualitatively and quantitatively analyzed and compared with *myo*-inositol hexaacetate as internal standard. The results showed that natural *C. sinensis* contained more than 7.99% free mannitol and a small amount of glucose, while its polysaccharides were usually composed of mannose, glucose and galactose with a molar ratio of 1.00:16.61~3.82:1.60~1.28. However, mannitol in cultured *C. sinensis* and cultured *C. militaris* were less than 5.83%, and free glucose was only detected in a few samples, while their polysaccharides were mainly composed of mannose, glucose and galactose with molar ratios of 1.00:3.01~1.09:3.30~1.05 and 1.00:2.86~1.28:1.07~0.78, respectively. Natural and cultured *Cordyceps* could be discriminated by hierarchical clustering analysis based on its free carbohydrate contents.

## 1. Introduction

Carbohydrates, including free and polymeric saccharides (polysaccharides), are usually considered the principal substrates of energy metabolism [1]. They also play important roles in nutrition and therapeutics [2,3]. In fact, fungi-derived polysaccharides have attracted a great deal of attention because of their significant pharmacological activities [4,5]. 

*Cordyceps*, a famous and valued medicinal material, is commonly used as a health food and Traditional Chinese Medicine for replenishing the kidneys and soothing the lungs in the treatment of various diseases [6,7]. It contains high amounts of carbohydrates, which can range from 3 to 8% of the total dry weight [8]. Most biological properties of *Cordyceps* can be attributed the presence of polysaccharides [9,10,11,12,13,14,15] which are regarded as potential markers for its quality control [8]. To date, though the sugars [16,17] and polysaccharides [18] in *Cordyceps* have been determined, complete profiles and contents of free and polymeric carbohydrates in natural and cultured *Cordyceps* are still not available.

Gas chromatography (GC) has been used as a powerful qualitative and quantitative tool for carbohydrate analysis [19,20,21]. Non-volatile carbohydrates can be converted to volatile derivatives, such as trimethylsilyl ether and acetate derivatives [22], amenable to GC analysis. In particular aldononitrile acetate derivatization is widely used due to its short sample preparation time, stable derivatized products and single chromatographic peak for each neutral saccharide [23], as well as its high reliability in qualitative and quantitative analysis [24,25,26,27,28]. The current study describes a method for the qualitative and quantitative determination of free carbohydrates and polysaccharides in natural and cultured *Cordyceps* using pressurized liquid extraction (PLE) and GC-MS. Their saccharide content characteristics were also compared.

## 2. Results and Discussion

### 2.1. Optimization of Derivatization

This operation involved two steps: oximation and acylation [28]. A previous study had shown that completed derivatization was markedly influenced by the amount of hydroxylamine hydrochloride used, the temperature and duration of oximation, as well as temperature and duration of acylation [29], hence these factors were investigated to obtain stable and maximum total response ratios of saccharides to internal standard (IS). 

**Figure 1:**
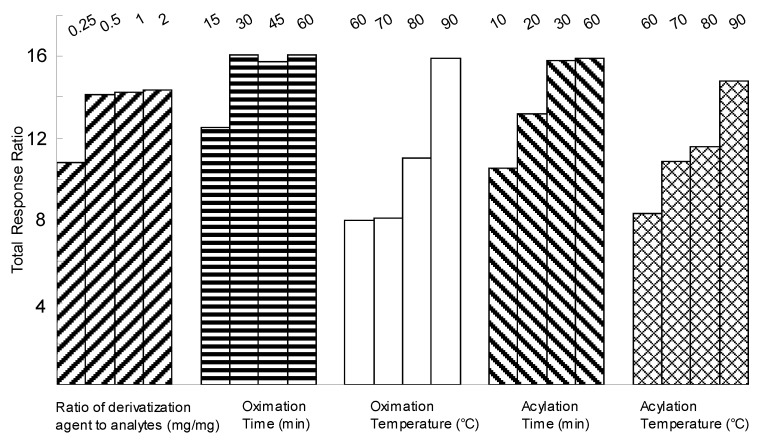
Optimization of derivatization of 10 investigated saccharides.

Due to fact that the presence of impurities could increase under high reaction temperatures, the temperature was optimized under 100 ºC. The results showed the optimum derivatization conditions involve mixing the analytes with a suitable ratio (mg/mg ≥ 1) of hydroxylamine hydrochloride and reacting at 90 ºC for 30 min, then acetic anhydride (50 μL per 1 mg of each analyte) is added to the cooled solution and the reaction continued at 90 ºC for 30 min (Figure 1).

### 2.2. Optimization of Trifluoroacetic Acid (TFA) Hydrolysis

**Figure 2:**
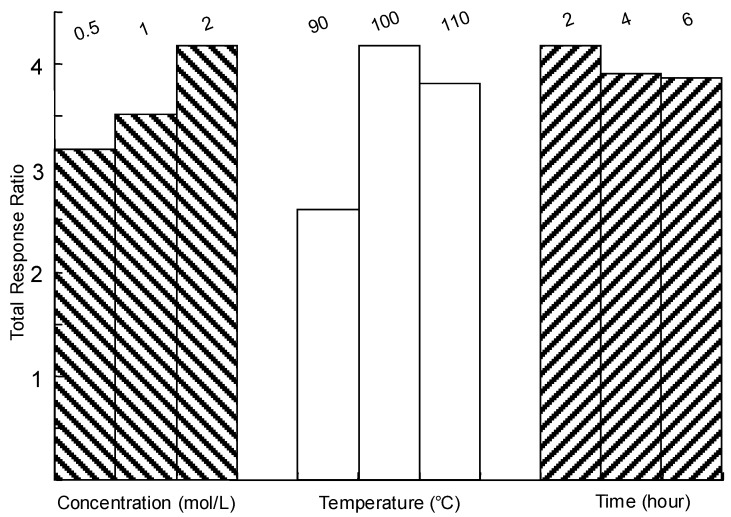
Optimization of TFA hydrolysis of polysaccharides.

TFA hydrolysis is a commonly used for hydrolysis of polysaccharides from *Cordyceps* [13,15]. To ensure complete release of compositional saccharides from the polysaccharides, a stable and maximum total response ratio of detected carbohydrates to IS was used as marker for evaluation of the hydrolysis efficiency. Figure 2 shows the effects of concentration of TFA, hydrolysis time and hydrolysis temperature on the total response ratio of the investigated saccharides. High concentration of TFA could induce the destruction of released saccharides and less hydrolysis time could result in incomplete hydrolysis. Finally, the optimized hydrolysis conditions were: concentration of TFA, 2 mol·L^-1^; temperature, 100 ºC; hydrolysis time, 2 h.

### 2.3. Optimization of Pressurized Liquid Extraction (PLE)

**Figure 3:**
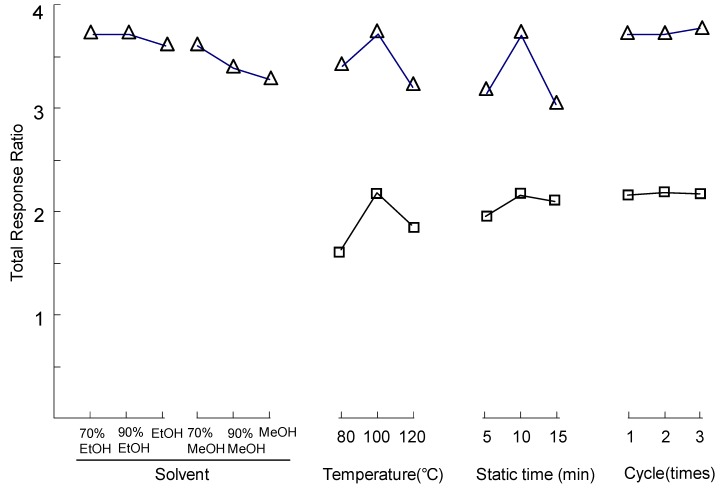
Optimization of pressurized liquid extraction of carbohydrates in *Cordyceps*. (∆) Free carbohydrates, (□) polysaccarides.

PLE is a rapid and effective method for sample preparation of carbohydrates [30,31]. Though monosaccharides and polysaccharides could be both extracted in one pressurized water extraction, miscalculation of conjugated carbohydrate contents could result because of the degradation of free carbohydrates in the samples during the acid hydrolysis of polysaccharides. Herein, a stepwise aqueous alcohol and water PLE method was applied for sample preparation based on the different polarity and solubility of the analytes in water. The parameters, including solvent, extraction temperature, static time and extraction cycle were optimized for complete extraction (Figure 3). The response ratio (peak area of analyte/peak area of IS) of free carbohydrates and polysaccaride were used for determination, respectively. The results showed that the optimized PLE parameters were: first, the free carbohydrates were extracted completely (no free sugars could be detected in the second－step water extract) by 70% aqueous ethanol, then the residue was used for the secondary water extract under 100 ºC to obtain polysaccharides; the static time was 10 min and one static cycle, pressure was set at default value (1.034 × 10^4^ kPa) for both steps.

### 2.4. Method Validation

The selected ion monitoring (SIM) method was applied for accurate determination of the saccharides. Characteristic fragment ions, *i.e.* *m/z* 129 for rhamnose, *m/z* 345 for fructose and sorbose, *m/z* 115 for seven other monosaccharides and IS, were selected for quantification (Table 1). The ketoses (fructose and sorbose) produced two peaks which represented their derivatives with *syn* and *anti* orientation [27], so the sum of peak area was used for their quantification. A series of amounts of stock solution containing 10 monosaccharides (from 60 μg to 2000 μg for each monosaccharide) were analyzed to establish calibration curves. The curves were constructed by plotting the response ratio *i.e.* the amount of each monosaccharide. Good linearities (R^2 ^> 0.9820) were obtained within the test ranges. The limits of quantification (LOQs) and detection (LODs) were 0.16–1.60 ng and 0.08–0.98 ng, respectively (Table 1). 

**Table 1:** Calibration data and LOQ, LOD for the derivatives of 10 monosacharides.

Analytes	SIM ( *m/z*)	Linear regression data			LOQ (ng)	LOD (ng)
		Regression equation	R^2^	Test range (ng)		
Rhamnose	129	y = 0.0407x - 0.0026	0.9996	0.96-31.94	0.33	0.08
Ribose	115	y = 0.0476x + 0.0283	0.9908	0.97-32.38	0.16	0.08
Arabinose	115	y = 0.0405x - 0.0025	0.9993	0.97-32.28	0.17	0.08
Xylose	115	y = 0.0421x - 0.0015	0.9995	0.96-32.12	0.45	0.08
Mannose	115	y = 0.0402x - 0.0068	0.9996	0.97-32.41	0.16	0.08
Glucose	115	y = 0.0361x - 0.0046	0.9997	0.96-32.04	0.16	0.13
Galactose	115	y = 0.0361x - 0.0095	0.9993	0.98-32.52	0.33	0.17
Mannitol	115	y = 0.0746x + 0.0038	0.9996	1.93-64.28	0.25	0.12
Fructose	345	y = 0.0512x - 0.1583	0.9820	3.26-32.65	1.60	0.98
Sorbose	345	y = 0.0598x - 0.1297	0.9927	1.61-32.18	1.29	0.88

The overall intra and inter-day variations of seven aldoses and mannitol were less than 5.19% (RSD%), and the accuracy was between 87.38% and 103.15%. Two ketoses (fructose and sorbose) gave the accuracy from 76.63% to 101.42% with RSD% between 1.56% and 18.04% (Table 2).

**Table 2:** Short and long term accuracy of the analytical method.

Analyte	Contain (ng)	Short term (n = 6)					Long term (n = 6)			
		Found (ng)	Accuracy(%) ^a^	RSD (%)			Found (ng)	Accuracy(%)	RSD (%)	
				Rt_R_^b^	RPa^c^				Rt_R_	RPa
Rhamnose	3.19	3.11	97.45	0.01	1.22		3.11	97.56	0.07	1.55
	15.97	15.65	98.17	0.02	1.40		15.55	97.58	0.06	1.38
	25.55	25.12	98.52	0.05	1.04		25.25	99.01	0.09	1.21
Ribose	3.24	2.82	87.38	0.02	0.97		3.11	88.36	0.06	1.27
	16.19	16.67	103.15	0.02	0.72		16.46	103.15	0.06	5.19
	25.91	24.28	93.89	0.05	1.11		24.27	93.88	0.11	1.03
Arabinose	3.23	3.12	96.86	0.01	0.79		3.13	97.14	0.07	1.07
	16.14	15.58	96.70	0.02	0.93		15.56	96.58	0.05	1.65
	25.82	25.58	99.21	0.04	0.83		25.71	99.74	0.10	0.62
Xylose	3.21	3.16	98.53	0.02	1.59		3.17	98.90	0.07	1.49
	16.06	15.68	97.79	0.04	0.84		15.61	97.42	0.07	1.05
	25.69	25.45	99.23	0.05	0.78		25.63	99.94	0.11	1.13
Mannose	3.24	3.13	96.86	0.01	1.42		3.17	97.92	0.06	1.52
	16.20	15.89	98.26	0.01	0.61		15.83	97.85	0.06	0.94
	25.93	25.58	98.84	0.03	0.95		25.63	99.04	0.07	0.47
Glucose	3.20	3.05	95.26	0.01	0.87		3.07	96.10	0.06	1.89
	16.02	15.77	98.64	0.03	0.80		15.70	98.18	0.05	1.91
	25.63	25.40	99.26	0.03	0.74		25.38	99.19	0.08	0.34
Galactose	3.25	3.06	94.38	0.01	1.13		3.08	95.01	0.06	1.46
	16.26	15.71	96.80	0.03	1.89		15.84	97.56	0.06	1.69
	26.02	25.85	99.54	0.03	1.05		25.93	99.84	0.08	0.06
Mannitol	6.43	6.20	96.64	0.01	1.24		6.20	96.69	0.03	1.39
	32.14	31.60	98.49	0.02	0.33		31.53	98.26	0.04	0.95
	51.42	51.14	99.62	0.02	0.58		51.15	99.64	0.06	0.41
Fructose	3.26	2.504	76.81	0.01	4.64		2.50	76.63	0.04	18.04
	16.33	16.39	100.56	0.01	2.39		14.85	91.12	0.03	8.76
	26.12	25.17	96.53	0.01	7.55		24.07	92.32	0.02	5.01
Sorbose	3.22	2.49	77.53	0.01	4.62		2.52	78.37	0.04	12.71
	16.09	16.29	101.42	0.01	1.56		15.51	96.57	0.03	4.91
	25.74	24.73	96.25	0.01	5.43		23.98	93.33	0.02	4.65

^a^ Accuracy (%) = 100 × mean of measured concentration/nominal concentration; ^b^ Rt_R_, relative retention time of analytes to internal standard (IS); ^c^ RPa, relative peak area ratio of analytes to IS.

Moreover, known amount of 10 monosaccharides were added into an accurately weighted sample (natural *C* *ordyceps sinensis* from Zhongqiao) for calculating recoveries. The mixture was extracted and analyzed using the method mentioned above. The overall recovery was range from 83.8%~104.5% for the analytes (Table 3). The results indicated the method is simple, sensitive and feasible for determination of investigated carbohydrates.

**Table 3:** Recoveries of 10 monosacharides for the analytical method.

Analyte	Original (ng)	Spiked (ng)	Found^ a ^(ng)	Recovery^ b^ (%)	RSD(%)
Rhamnose	－^c^	3.83	3.54	92.3	4.7
Ribose	－	3.89	3.61	93.0	3.9
Arabinose	－	3.87	3.67	94.7	5.6
Xylose	－	3.85	3.68	95.4	3.2
Mannose	－	3.89	4.06	104.5	3.4
Glucose	1.78	3.84	5.72	102.4	4.6
Galactose	－	3.90	3.76	96.3	6.1
Mannitol	34.85	7.71	41.97	92.2	4.6
Fructose	－	3.92	3.28	83.8	7.0
Sorbose	－	3.86	3.43	88.9	9.3

^a^ The data was present as average of three determinations; ^b^ Recovery (%) = 100×(amount found-original amount)/amount spiked; ^c^ Undetectable.

### 2.5. Compositional Sugars in Polysaccharides

**Figure 4:**
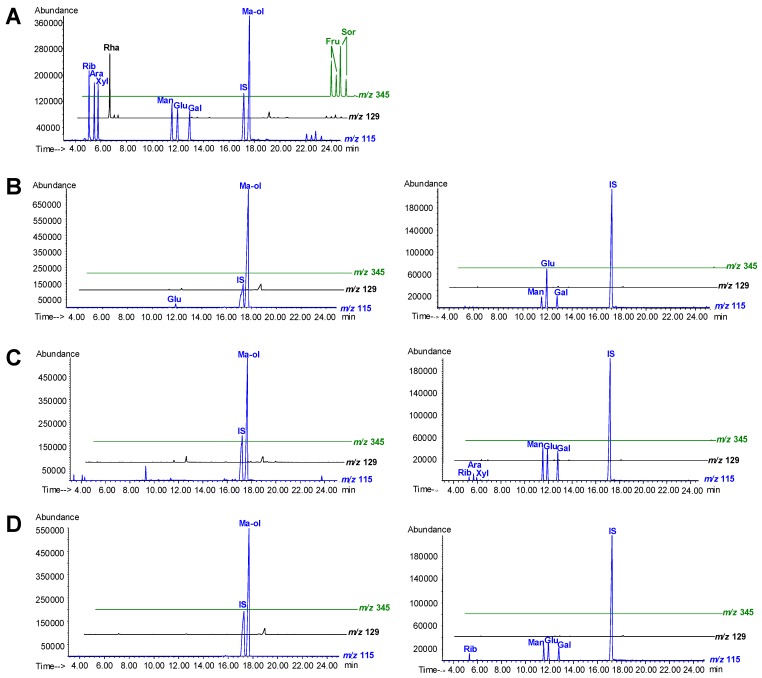
Typical SIM chromatograms of mixed standards (A), free (left) and conjunct carbohydrates (right) in natural *Cordyceps sinensis* (B), cultured *C. sinensis* (C), cultured *C. militaris* (D). Rha, rhamnose; Rib, ribose; Ara, arabinose; Xyl, xylose; Man, mannose; Glu, glucose; Gal, galactose; Ma-ol, mannitol; Fru, fructose; Sor, sorbose; IS, internal standard.

Next the molar ratio value of released monosacchrides in the polysaccharides was determined after hydrolysis using known amounts of authentic monosaccharide standards as reference. The results are shown in Table 4. Polysaccharides in natural *C. sinensis* (NCS) were usually composed of mannose, glucose and galactose with a molar ratio of 1.00:16.61~3.82:1.60~1.28. Otherwise, the compositional saccharides and their molar ratios of cultured *C. sinensis* (CCS) and cultured *C. militaris* (CCM) were 1.00:3.01~1.09:3.30~1.05 (except the Hongkong sample) and 1.00:2.86~1.28:1.07~0.78, respectively, besides, some samples contained traces of ribose, arabinose and xylose. Generally, more types of saccharides were found in polysaccharides of cultured *Cordyceps* than in those of natural *Cordyceps* (Figure 4).

**Table 4:** Molar ratio of compositional sugars of polysaccharides in natural and cultured *Cordyceps*.

Samples	Ribose	Arabinose		Xylose	Mannose	Glucose	Galactose
**Natural *C. sinensis***							
Zhongqiao					1.00	16.61	1.28
Sichuan					1.00	3.82	1.40
Qinghai					1.00	4.33	1.31
Tibet					1.00	13.65	1.60
**Cultured *C. sinensis***							
Wanfeng		0.11		0.13	1.00	1.97	1.36
Anhui	0.02	0.14		0.07	1.00	1.09	1.11
Hebei	0.24			0.11	1.00	2.60	3.03
HongKong					1.00	25.40	0.46
Huadong	0.05				1.00	3.01	1.05
Jiangxi		0.32		0.15	1.00	1.18	1.96
**Cultured *C. militaris***							
Aoli					1.00	2.86	0.78
Xiankang	0.01				1.00	2.17	0.93
Quanxin	0.14				1.00	1.28	1.07

### 2.6. Quantitation of Free Carbohydrates

The contents of free carbohydrates were the values of measuring 70% ethanol extracts after direct derivatization. Data were summarized in Table 5. NCS contained the highest amounts of mannitol (≥7.99%) and a little amount of glucose. However, mannitol in both cultured CCS and cultured CCM were less than 5.83%, and free glucose was only detected in few samples (CCS from HongKong, CCM from Aoli and Xiankang). The displayed distinction between natural and cultured *Cordyceps* is according with previous reports [8]. 

**Table 5:** Contents of free and conjunct carbohydrates (mg per 100 mg) in natural and cultured *Cordyceps*.

Analyte		Natural *C. sinensis*					Cultured C. sinensis							Cultured *C. militaris*		
		Zhongqiao	Sichuan	Qinghai	Tibet		Wanfeng	Anhui	Hebei	HongKong	Huadong	Jiangxi		Aoli	Xiankang	Quanxin
Rhamnose	F^a^	－^b^	－	－	－		－	－	－	－	－	－		－	－	－
	C	－	－	－	－		－	－	－	－	－	－		－	－	－
Ribose	F	－	－	－	－		－	－	－	－	－	－		－	－	－
	C	－	－	－	－		－	±	0.02	－	±	－		－	±	±
Arabinose	F	－	－	－	－		－	－	－	－	－	－		－	－	－
	C	－	－	－	－		±	±	－	－	－	0.23		－	－	－
Xylose	F	－	－	－	－		－	－	－	－	－	－		－	－	－
	C	－	－	－	－		±	±	±	－	－	±		－	－	－
Mannose	F	－	－	－	－		－	－	－	－	－	－		－	－	－
	C	0.15^c^	0.19	0.24	0.25		0.60	0.72	0.42	0.20	0.46	0.89		0.24	0.28	0.32
Glucose	F	0.29	±^d^	±	0.13		－	－	－	±	－	－		±	0.13	－
	C	2.51	0.73	1.03	3.37		1.17	0.79	1.09	5.03	1.39	1.05		0.70	0.60	0.41
Galactose	F	－	－	－	－		－	－	－	－	－	－		－	－	－
	C	0.19	0.27	0.31	0.39		0.81	0.80	1.27	±	0.48	1.74		0.19	0.26	0.34
Mannitol	F	8.14	9.76	7.99	10.74		5.83	4.01	4.44	0.94	2.24	4.30		3.16	4.09	3.96
Fructose	F	－	－	－	－		－	－	－	－	－	－		－	－	－
	C	－	－	－	－		－	－	－	－	－	－		－	－	－
Sorbose	F	－	－	－	－		－	－	－	－	－	－		－	－	－
	C	－	－	－	－		－	－	－	－	－	－		－	－	－

^a^ F, free carbohydrates; C, polysaccarides; ^b^ Undetectable; ^c^ Average of duplicates; ^d^ Under the limits of quantitation.2.7. Quantitation of Conjunct Carbohydrates

### 2.7. Quantitation of Conjunct Carbohydrates

The contents of conjunct carbohydrates were the values obtained by measuring water extracts after TFA hydrolysis and derivatization. After hydrolysis, the contents of glucose increased significantly in all of samples, and galactose and mannose could also be calculated (Table 5). The glucose released from polysaccharides in *C. sinensis* (0.73~5.03%) was higher than that in *C. militaris* (0.41–0.70%), while galactose and mannose found in polysaccharides from NCS, CCS and CCM were 0.19–0.39% and 0.15–0.25%, 0.48–1.74% and 0.42–0.89%, as well as 0.19–0.34% and 0.24–0.32%, respectively. Besides, trace ribose, arabinose and xylose could be mainly found in CCS, while only trace ribose contained in CCM. More types of saccharides contained in CCS might derive from the culture media, which need further investigation.

**Figure 5:**
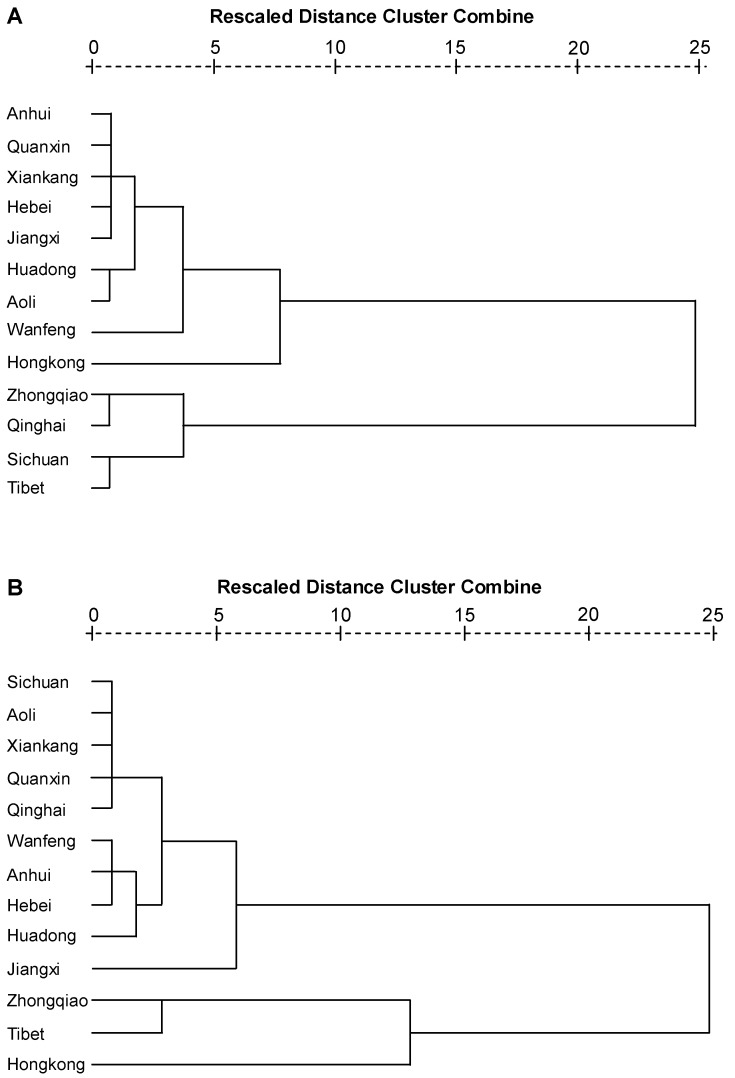
Dendrograms of hierarchical cluster analysis resulting from the content of free (A) and conjunct (B) carbohydrates in 13 samples of *Cordyceps*.

### 2.8. Comparison of natural and cultured Cordyceps

Hierarchical cluster analysis was preformed depend on the content of free saccharides and conjunct carbohydrates (Figure 5). The results showed natural and cultured *Cordyceps* could be grouped into two clusters, which indicated the carbohydrates are helpful for the characterization. However, there were no clear clusters to discriminate NCS, CCS and CCM, perhaps because the total contents had no significant differences, even though the types of carbohydrates contained in the polysaccharides varied

## 3. Experimental Section

### 3.1. Materials and Chemicals

Natural *C. sinensis* were obtained from Sichuan, Tibet, Qinghai provinces and Zhongqiao Herbal Store in Macau (originally from Tibet); Cultured *C. sinensis* were obtained from Wanfeng (Wanfeng Medicines Group Co. Ltd., Zhejiang Province), Anhui (Anhui Agricultural University, Anhui Province), Hebei (Boding Pharmaceutical Factory, Hebei Province), Hongkong (The Hong Kong University of Science and Technology, Hongkong), Huadong (Huadong Medicines Group Co. Ltd., Zhejiang Province) and Jiangxi (Chinese Medicine Factory of Jiangxi, Jiangxi Province); Cultured *C. militaris*, one of the substitutes of *C. sinensis*, were obtained from Aoli (Zhangjiagang Aoli Bio-health Products Co. Ltd., Jiangsu Province), Xiankang (Zhuhai Xiankang Bio-Tech Co. Ltd., Guangdong Province) and Quanxin (Quanxin Company, Malaysia). The botanical origin of materials was identified by corresponding author and the voucher specimens were deposited at the Institute of Chinese Medical Sciences, University of Macau, Macau, China. L-Rhamnose, D-ribose, D-arabinose, D-xylose, D-mannose, D-glucose, D-galactose, D-mannitol, D-fructose and L-sorbose were purchased from Sigma (St. Louis, MO, USA). A stock solution containing mannitol (~2 mg·mL^-1^) and other nine monosaccharides (~1 mg·mL^-1^ for each) was prepared in pyridine (ReagentPlus, ≥99%, Sigma-Aldrich). A solution containing hydroxylamine hydrochloride (analytically pure, ~20 mg·mL^-1^) was prepared in pyridine for derivatization of saccharides. About 100 mg *myo*-inositol hexaacetate (prepared according to the reference [29], >99% as determined by GC-MS) was dissolved in chloroform (25 mL) as an IS solution. These solutions were all stored at 4 ºC. Trifluoroacetic acid (TFA, 99%) was purchased from Riedel-de Haën (Seelze, Germany). Acetic anhydride and chloroform were analytical grade reagents. Water was obtained from a Millipore water-purification system (Millipore, Bedford, MA, USA).

### 3.2. Pressurized Liquid Extraction

PLE was performed on a Dionex ASE 200 system (Dionex Corp., Sunnyvale, CA, USA) in two steps under optimized conditions. Dried powder of *Cordyceps* (~0.1 g) was mixed with diatomaceous earth in a proportion 1:1 and placed into an 11 mL stainless steel extraction cell, then extracted with 70% aqueous ethanol under 100 ºC for 10 min of static time for 1 cycle with pressure at 1.034 × 10^4^ kPa. The extract purged out by nitrogen was transferred into a 25 mL volumetric flask, which was made up to its volume with the bsame solvent for the analysis of free carbohydrates. The residue was further extracted with water under 100 ºC for 10 min of static time for 1 cycle with pressure at 1.034 × 10^4^ kPa. The extract was also transferred into a 25 mL volumetric flask which was made up to its volume with water for the analysis of conjunct carbohydrates.

### 3.3. TFA Hydrolysis

An aliquot (10 mL) of PLE water extract was evaporated to dryness, then hydrolyzed with 2 mol·L^-1^ TFA (1 mL) in a sealed glass tube with screw cap which filled with pure nitrogen gas at 100 ºC for 2 h. The hydrolyzed solution was evaporated to dryness under 45 ºC and then methanol (1 mL) was added for further evaporation and complete removal of TFA. The hydrolysate was used for derivatization.

### 3.4. Derivatization

For standard monosaccharides, stock solution (1 mL) was treated with hydroxylamine hydrochloride-pyridine solution (1 mL) in a sealed glass tube with a screw cap at 90 ºC for 30 min. After cooling to room temperature, acetic anhydride (1 mL) was added and heating continued for another 30 min in the resealed tube. The cooled solution was evaporated to dryness under diminished pressure at 45 ºC. The residue was dissolved in dry chloroform (2 mL) and IS solution (500 μL) was added. The mixture was filtered through 0.45 μm syringe filter (Agilent Technologies) prior to injection into GC-MS system. For free carbohydrates and polysacarides, an aliquot (10 mL) of the extracts was evaporated to dryness. The residue was reacted with hydroxylamine hydrochloride and acetic anhydride to form the derivatives directly as in the procedures mentioned above for free carbohydrate determination. The dried residue of acidic hydrolyzed polysaccharides was derivatized for polysaccaride determination.

### 3.5. GC-MS Analysis

GC-MS was performed on an Agilent 6890 gas chromatography instrument coupled with an Agilent 5973 mass spectrometer (Agilent Technologies, Palo Alto, CA, USA). A HP-5MS capillary column (30 m × 0.25 mm, i.d.) coated with 0.25 μm film 5% phenyl methyl siloxane was used for separation. The column temperature was set at 175 ºC and held for 7 min, then programmed at 5 ºC·min^-1^ to 185 ºC and held for 5 min, then at 4 ºC·min^-1^ to 230 ºC. Split injection (2 μL) with a split ratio of 1:50 was applied. High purity helium was used as carrier gas with flow rate of 1.0 mL·min^-1^. The mass spectrometer was operated in electron-impact (EI) mode, the scan range was 40–550 amu, the ionization energy was 70 eV and the scan rate was 2.89 s per scan. The inlet, ionization source temperature were 250 and 280 ºC, respectively.

### 3.6. Data Analysis

Hierarchical cluster analysis was performed by SPSS 13.0 for Windows (SPSS Inc., Chicago, IL, USA), which comprise a number of “procedures” – graphical, statistical, reporting, processing and tabulating procedures – that enable simple and rapid data evaluation. Average Linkage between groups was selected as measurement for hierarchical clustering analysis.

## 4. Conclusions

Free carbohydrates and polysaccharides in natural and cultured *Cordyceps* were qualitatively and quantitatively determined using a stepwise pressurized liquid extraction and gas chromatography coupled with mass spectrometry. Natural and cultured *Cordyceps* could also be discriminated by hierarchical clustering analysis based on the contents of free carbohydrates. It is indicated the carbohydrates could be characteristic for evaluation of *Cordyceps*, and the established method is helpful for elucidating the distinct carbohydrates in samples of different origin. 
